# Management of oral anticoagulation after cardioembolic stroke and stroke survival data from a population based stroke registry (LuSSt)

**DOI:** 10.1186/s12883-014-0199-7

**Published:** 2014-10-08

**Authors:** Frederick Palm, Martin Kraus, Anton Safer, Joachim Wolf, Heiko Becher, Armin J Grau

**Affiliations:** Department of Neurology, Städtisches Klinikum Ludwigshafen, Bremserstr. 79, 67063 Ludwigshafen, Germany; Department of Internal Medicine, Klinikum, Ludwigshafen, Germany; Institute of Public Health, Universitätsklinikum Heidelberg, Heidelberg, Germany

**Keywords:** Oral anticoagulation, Cardioembolic stroke, Atrial fibrillation

## Abstract

**Background:**

Cardioembolic stroke (CES) due to atrial fibrillation (AF) is associated with high stroke mortality. Oral anticoagulation (OAC) reduces stroke mortality, however, the impact of OAC-administration during hospital stay post ischemic stroke on mortality is unclear. We determined whether the timing of OAC initiation among other prognostic factors influenced mortality after CES.

**Methods:**

Within the Ludwigshafen Stroke Study (LuSSt), a prospective population-based stroke register, we analysed all patients with a first ever ischemic stroke or TIA due to AF from 2006 until 2010. We analysed whether treatment or non-treatment with OAC and initiation of OAC-therapy during and after hospitalization influenced stroke mortality within 500 days after stroke/TIA due to AF.

**Results:**

In total 479 patients had a first-ever ischemic stroke (n = 394) or TIA (n = 85) due to AF. One-year mortality rate was 28.4%. Overall, 252 patients (52.6%) received OAC. In 181 patients (37.8%), OAC treatment was started in hospital and continued thereafter. Recommendation to start OAC post discharge was given in 110 patients (23.0%) of whom 71 patients received OAC with VKA (14.8%). No OAC-recommendation was given in 158 patients (33.0%). In multivariate Cox regression analysis, higher age (HR 1.04; 95% CI 1.02-1.07), coronary artery disease (HR: 1.6; 95% CI 1.1-2.3), higher mRS-score at discharge (HR 1.24; 95% CI 1.09-1.4), and OAC treatment ((no OAC vs started in hospital (HR: 5.4; 95% CI 2.8-10.5), were independently associated with stroke mortality. OAC-timing did not significantly influence stroke mortality (started post discharge vs. started in hospital (HR 0.3; 95% CI 0.07-1.4)).

**Conclusions:**

OAC non-treatment is the main predictor for stroke mortality. Although OAC initiation during hospital stay showed a trend towards higher mortality, early initiation in selected patients is an option as recommendation to start OAC post hospital was implemented in only 64.5%. This rate might be elevated by implementation of special intervention programs.

## Background

Atrial fibrillation (AF) is the main cause of cardioembolic stroke (CES), the dominant ischemic stroke subtype in the elderly [1,2]. Additionally, non-diagnosed AF is likely to be responsible for many cryptogenic strokes [3]. Prevalence of AF increases with age and lifetime risk of developing AF is estimated to be 25% after reaching the age of 40 [4]. Due to demographic changes in industrial countries, prevalence of AF is predicted to increase in the future [5]. Concomitant to the incidence of AF, incidence of CES is likely to increase. CES is associated with higher stroke severity and mortality [6,7]. Stroke recurrence is associated with much worse outcome [6,7]. Early stroke recurrence is frequent in ischemic stroke due to large artery atherosclerosis [8], in contrast, it is less common in CES [8,6]. However, recurrence risk of CES is highest among stroke subtypes in the long term [7]. Oral anticoagulation (OAC) with vitamin K antagonists (VKA) and new oral anticoagulants (NOAC) is effective in primary and secondary stroke prevention and in reducing mortality [9,10]. Both, VKAs and NOACs increase risk of intracerebral hemorrhage (ICH) [11]. As early hemorrhagic transformation occurs in CES in up to 17% within 5 days [12], and occurence of ICH is reported to be up to 12% [13], timing of OAC initiation after acute CES is still a controversial issue and its impact on mortality is not clear.

We hypothesized that initiation of OAC in hospital after first-ever CES is associated with a significant reduction on mortality compared to OAC non-treatment and OAC initiation post discharge. Implementation of OAC recommendation was additionally analysed.

## Methods

The “Ludwigshafen Stroke Study” (LuSSt) is a population-based, prospective registry of stroke and transient ischemic attack (TIA) in the City of Ludwigshafen (Germany), starting January 1st, 2006. A detailed description of LuSSt has been published recently [14]. All patients with first-ever ischemic strokes (FEIS) due to AF until December 31st, 2010 were included in the present analysis.

### Summary of study population, case ascertainment follow-up and standard definitions

Ludwigshafen is an industrial city in the state of Rhineland-Palatinate in Western Germany. The total source population was 167,657 inhabitants (83,009 males and 84,648 females) on December 31st, 2008, which was the midpoint of the study period. Multiple overlapping methods of patient identification were used in order to achieve complete case ascertainment as described before [14]. Collaboration with all hospitals in Ludwigshafen and surrounding hospitals treating stroke patients outside the city boundaries ensured complete case acquisition. Patients who have been treated in other hospitals, hospitals abroad and non-hospitalized patients were identified by contacting all general practitioners, specialists in internal medicine and neurologists practicing in Ludwigshafen. In addition nursing and residential homes were contacted regularly. In case of identified stroke patients via death certificate, patients’ general practitioner was contacted in order to achieve more information, especially with regards to stroke symptoms, current stroke and antithrombotic treatment. Follow up investigations were conducted by telephone 28 days, 3, 12 and 36 months after stroke onset, using a standardised questionnaire, if written informed had been given by patients or their legal representatives. Information about survival was collected by population registration authority in all patients without statement of consent, or if patients could not be contacted. The study (LuSSt) was approved by the ethics committee of “Landesärztekammer Rhineland-Palatinate” (reference number: 837.333.05) and the local data protection commissioner of Rhineland-Palatinate. Stroke was defined according to the definition of the World Health Organization (WHO) [15]. Stroke subtype classification was based on the results of brain imaging, discriminating between ischemic stroke (IS), intracerebral hemorrhage (ICH) or subarachnoid hemorrhage (SAH). Patients with no clinical evidence of any previous stroke event were diagnosed as first-ever stroke (FES) irrespective of brain imaging. Assignment to etiology in ischemic stroke was performed using modified TOAST-criteria and has been described in detail before [2]. TIA was defined as a transient focal cerebral ischemia with symptoms lasting <24 hours independent of neuroimaging results. Due to current changes in the definition of TIA by usage of MRI imaging, we included patients with both, IS and TIA. All patients admitted to the Klinikum Ludwigshafen received intensive diagnostic workup as previously described [2]. About 90% of all patients with FES in the source population are admitted to this hospital.

### Neuroradiological findings, risk factors, risk stratification scores and stroke severity

Neuroradiological findings were categorized as territorial infarction (TI), lacunar infarction (LI), hemorrhagic transformation (HT) including hemorrhagic infarction, defined as petechial bleeding in the infarction area, and parenchymal hematoma as hematoma in the infarcted area with a space-occupying effect or a traumatic subarachnoid hemorrhage (SAH) due to ischemic infarction [12]. Hemorrhagic transformation was detected in CT/MRI performed on admission or in repeated CT/MRI performed during hospital stay. Cardiovascular risk factors were defined according to current national and international guidelines as recently described in detail [2]. AF (known or new diagnosis) was defined with current ESC guidelines as irregular RR intervals with a variable atrial cycle length and absent P waves in the surface ECG [16]. Other sources of cardiac embolism have been ruled out by using transthoracic or transesophageal echocardiography. The CHA_2_DS_2_-VASc and HASBLED-Score were used as risk stratification tools to evaluate stroke and bleeding risk [17]. Stroke severity was analysed using the National Institute of Health Stroke Scale (NIHSS) on admission and modified Rankin-Scale (mRS) on discharge by experienced neurologists of the study team [18,19]. The glomerular filtration rate was determined on admission using Modification of Diet in Renal Disease (MDRD) formula [20].

### Antithrombotic treatment

Prestroke treatment with VKA (=oral anticoagulation) and antiplatelet agents was assessed. In patients receiving VKA, international normalized ratio (INR) was determined on admission. Poststroke management was subdivided as follows: 1. No OAC (neither given nor recommended and OAC recommended but not given post hospital stay); 2. OAC started in hospital; 3. OAC started post discharge; 4. Unknown. If OAC was started in hospital or recommended and status post hospital was unknown, OAC management post stroke was ascertained unknown. If VKA treatment had not been recommended, reasons were protocolled. Posthospital treatment with VKA after 30 days, as well as 3, 12 and 36 month after stroke were checked. In order to include majority of patients in univariate and multivariate Cox regression analysis, vital status was additionally assessed 500 days after stroke onset. Recurrent stroke, TIA or bleeding were documented within 500 days after stroke.

### Statistical analyses

*Χ*^2^-test was used to compare categorical data and fisher exact test was applied to analyse normally distributed continuous data in univariate analysis. All variables being significant except risk stratification scores were then included in a multivariate Cox regression model followed by backward elimination of nonsignificant variables (p < 0,05). In order to lower impact of stroke severity on stroke mortality, patients who died within 7 days after stroke were excluded for multivariate Cox regression analysis. All data were analysed using SAS 9.3 software (SAS Institute, North Carolina, RRID:nif-0000-31484). All tests were two-sided. Level of significance was set to 5% for all tests.

## Results

During study period, 502 patients had a FEIS or TIA due to AF. ICH was ruled out in all patients by neuroimaging. As survival status was unknown in 23 patients (4.8%), 479 patients with FEIS (n = 394) and TIA (n = 85) were finally included in the present analysis. Mean age was 79.3 years +/− 10.1 (women: 80.7 years +/− 10.5; men: 77.4 years +/− 9.1). Median of hospital stay was 10.8 days (range 1–35 days). 332 patients (69.3%) were initially admitted to a stroke unit, whereas 29 patients (6.1%) received intensive care treatment. Overall, 18 patients (3.8%) were treated ambulatory. Thirteen patients died within first week. One year mortality rate was 28.4% and mortality rate after 500 days was 30.7%.

**Figure 1:**
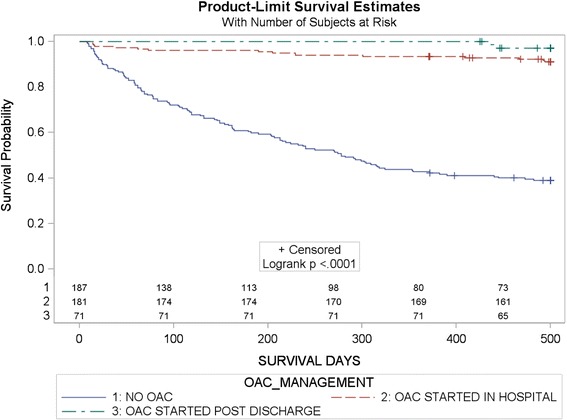
**Kaplan-Meier estimate of survival after ischemic stroke according to OAC management ((n = 439) patients who died within 7 days (n = 13) excluded).**

Neuroimaging was performed in all patients with 106 patients (22.1%) receiving cranial MRI. AF was newly diagnosed in 62 (12.9%) patients. Acute ischemic tissue damage could be observed in 20 patients (23.5%) with TIA based on neuroimaging. Due to study period, no patient received NOAC treatment. Baseline characteristics of all patients are displayed in Tables 1 and 2. Overall, OAC with VKA was started in hospital in 184 patients and it was continued post hospital in 181 patients (98.4%). Starting OAC post discharge was recommended in total in 130 patients. In 20 out of these patients OAC status post discharge was unknown and OAC-recommendation was implemented in 71 patients (64.5%). Figure 1 shows Kaplan-Meier estimate of survival after ischemic stroke according to OAC management. Antiplatelet therapy was prescribed in 173 (36.1%) patients. There was no patient who received a combination of OAC and antiplatelet agent. Main reasons for non-OAC recommendation were bad general condition (28%, n = 41), risk of falling (27%, n = 39) and dementia (13%, n = 19). As compared to those patients with recommendation to start OAC treatment post discharge, patients receiving OAC treatment in hospital had signficant lower initial stroke severity as indicated by NIHSS (p > 0.0001), lower HASBLED-score (p = 0.001), less signs of hemorrhagic transformation (p = 0.002) and territorial infarction (p > 0.0001), high rate of premedication with VKA (p < 0.0001), low rate of premedication with antiplatelets (p < 0.0001) and significant lower mRS-score (p < 0.0001) on discharge. Recurrent stroke within 500 days occurred in 43 patients (9%) with 13 patients (30,2%) being on OAC (started in hospital: n = 8 (61.5%) vs. started post discharge: n = 5 (38.5%), of which 6 patients (46.1%) had an INR-value below 2. Three out of 5 patients (60%) with OAC having an INR > 2, had a recurrent ischemic stroke within 30 days after OAC. One year recurrence rate was 7.1% (n = 34), whereas rate of early stroke recurrence within 14 days was 0.8% (n = 4) and within 7 days 0.4% (n = 2). OAC associated ICH was observed in three patients (0.6%), two (66.7%) occuring within 30 days after initial event.

**Table 1:** **Baseline characteristics of 479 patients with AF-associated FE-IS and TIA (neuroimaging, stroke severity and risk stratification scores)**

Variable (N*)		**Death within 500 days**	**Survival at 500 days**	**p-value univariate**	**p-value multivariate**
	N (%)				
**Gender **(479*/479)				p=0.31	
Male	197 (41.1%)	55 (27.9%)	142 (72.1%)		
Female	282 (58.9%)	92 (32.6%)	190(67.4%)		
**Age (years)** (479*/479)				**p<0.0001**	**p=0.001**
20-65	44 (9.2%)	3 (6.8%)	41 (93.2%)		
66-75	105 (21.9%)	18 (17.1%)	87 (82.9%)		
76-85	204 (42.6%)	56 (27.5%)	148 (72.6%)		
>85	126 (26.3%)	70 (55.6%)	56 (44.4%)		
**Diagnosis **(479*/479)				**p=0.0001**	p=0.95
Ischemic stroke	394 (82.3%)	135 (34.3%)	259 (65.7%)		
TIA	85 (17.7%)	12 (14.1%)	73 (85.9%)		
**Neuroradiological Findings **(476*/479)					
Territorial infarction (recent)	256 (53.8%)	99 (38.7%)	157 (61.3%)	**p<0.0001**	***p=0.055***
Territorial infarction (old)	45 (9.5%)	15 (33.3%)	30 (66.7%)	p=0.61	
Hemorrhagic transformation	26 (5.5%)	14 (53.8%)	12 (46.2%)	**p=0.01**	p=0.46
Hemorrhagic infarction	15 (57.7%)	7 (46.7%)	8 (53.3%)		
Parenchymal haematoma	8 (30.8%)	6 (75%)	2 (25%)		
Subarachnoidal hemmorh.	3 (11.5%)	1 (33.3%)	2 (66.7%)		
**Stroke severity**					
*NIHSS at admission (*461*/479)				**p<0.0001**	p=0.8
0-3	227 (49.2%)	38 (16.7%)	189 (83.3%)		
4-8	117 (25.4%)	39 (33.3%)	78 (66.7%)		
>9	117 (25.4%)	62 (53%)	55 (47%)		
*mRS at discharge (*470*/479)				**p<0.0001**	**p=0.0007**
0-1	197 (41.9%)	23 (11.7%)	174 (88.3%)		
2-3	123 (26.2%)	38 (30.9%)	85 (69.1%)		
>4	150 (31.9%)	85 (56.7%)	65 (43.3%)		
**Risk stratification score**					
*CHA*_*2*_ *DS*_*2*_ *-Vasc-Score (*452*/479)				**p=0.0003**	
2-4	30 (6.6%)	2 (6.7%)	28 (93.3%)		
5	93 (20.6%)	23 (24.7)	70 (75.3%)		
6	159 (35.2%)	42 (26.4%)	117 (73.6%)		
7	95 (21%)	31 (32.6%)	64 (67.4%)		
8-9	75 (16.6%)	35 (46.7%)	40 (53.3%)		
*HASBLED-Score (*458*/479)				**p<0.0001**	
0-2	115 (25.1%)	18 (15.7%)	97 (84.8%)		
3	301 (65.7%)	100 (33.2%)	201 (66.8%)		
4	39 (8.5%)	18 (46.2%)	21 (53.6%)		
5	3 (0.7%)	2 (66.7%)	1 (33.3%)		

*Patients with complete information.

Significant p-values in bold.

**Table 2:** **Baseline characteristics of 479 patients with AF-associated FE-IS and TIA (risk factors and treatment)**

Variable (N*)		**Death within 500 days**	**Survival of 500 days**	**p-value univariate**	**p-value multivariate**
	N (%)				
**Risk factors**					
CAD (465*/479)	144 (31%)	54 (37.5%)	90 (62.5%)	**p=0.02**	**p=0.01**
MI (475*/479)	84 (17.7%)	33 (39.3%)	51 (60.7%)	p=0.07	
Heart failure (452*/479)	169 (37.4%)	62 (36.7%)	107 (63.3%)	**p=0.01**	p=0.94
PAD (461*/479)	48 (10.4%)	23 (47.9%)	25 (52.1%)	**p=0.004**	p=0.13
Hypertension (479*/479)	446 (93.1%)	136 (30.5%)	310 (69.5%)	p=0.70	
Diabetes (478*/479)	157 (32.8%)	43 (27.4%)	114 (72.6%)	p=0.34	
Hypercholesterolemia (470*/479)	285 (60.6%)	79 (27.7%)	206 (72.3%)	p=0.26	
Smoking (400*/479)				p=0.24	
Nonsmoker	206 (51.5%)	69 (33.5%)	137 (66.5%)		
Smoker	42 (10.5%)	11 (26.2%)	31 (73.8%)		
Former smoker	152 (38%)	39 (25.7%)	113 (74.3%)		
**GFR **(461*/479)				**p<0.0001**	p=0.09
0-<30	16 (3.5%)	8 (50%)	8 (50%)		
30-<60	160 (34.7%)	66 (41.3%)	94 (58.7%)		
≥60	285 (61.8%)	65 (22.8%)	220 (77.2%)		
**Treatment **(479*/479)					
*Intraveneous thrombolysis*				p=0.62	
*Yes*	47 (9.8%)	16 (34%)	31 (66%)		
*No*	432 (90.2)	131 (30.3)	301 (69.7)		
*Antithrombotic treatment (*479*/479)					
OAK prior to stroke	116 (24.2%)	20 (17.2%)	96 (82.8%)	**p=0.0003**	p=0.91
INR <2	65 (58%)	15 (23.1%)	50 (76.9%)		
INR ≥2	44 (39.3%)	4 (9.1%)	40 (90.9%)		
INR unknown	7 (6%)	1 (14.3%)	6 (85.7%)		
Antiplatelets prior to stroke (479*/479)				**p<0.0001**	
No antiplatelet	298 (62.2%)	69 (23.2%)	229 (76.8%)		
Antiplatelet	181(37.8%)	78 (43.1%)	103 (56.9%)		
OAC management post stroke (479*/479)				**p<0.0001**	**p<0.0001**
No VKA	200 (41.8%)	127 (63.5%)	73 (37.5%)		
OAC neither given, nor recommended	158 (79%)	109 (69%)	49 (31%)		
OAC recommended, but not given	42 (21%)	18 (42.9%)	24 (57.1%)		
VKA started in hospital	181 (37.8%)	16 (8.8%)	165 (91.2%)		
VKA started post discharge	71 (14.8%)	2 (2.8%)	69 (97.2%)		
Unknown	27 (5.6%)	2 (7.4%)	25 (93.6%)		
**Recurrent cerebral event (500days)**	43/479 (9%)	21 (48.8%)	22 (51.2%)	**p=0.009**	p=0.27
IS	33 (76.7%)	19 (57.6%)	14 (42.4%)		
TIA	6 (14%)	0	6		
ICH	4 (9.3%)	2 (50%)	2 (50%)		

*Patients with complete information.

Significant p-values in bold.

**Figure 2:**
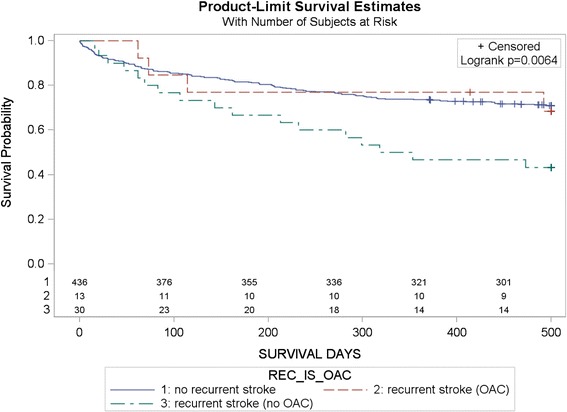
**Kaplan-Meier estimate of survival after ischemic stroke according to recurrence of stroke on and off OAC treatment (n = 479).**

Results of Cox regression analysis are displayed in Table 3. Higher age, higher mRS-score at discharge, diagnosis of CAD, and OAC treatment post stroke were independently associated with stroke mortality. Figure 2 additionally presents Kaplan-Meier estimate of survival with regard to stroke recurrence. Poststroke survival was highest in those patients receiving OAC irrespective of stroke recurrence.

**Table 3:** **Multivariate Cox regression analysis**

***Variable***	**HR (95% CI)**	**P-value**
***Model A n = 397 (90.4%)***		
Age (per year)	1.04 (1.02-1.07)	**p = 0.001**
CAD (Yes vs. No)	1.6 (1.1-2.3)	**p = 0.01**
mRS at discharge (per year)	1.2 (1.1-1.4)	**p = 0.0007**
OAK management post stroke (VKA in hospital as reference)		
*No VKA*	5.4 (2.8-10.5)	**p < 0.0001**
*VKA (started post discharge)*	0.3 (0.07-1.4)	**p = 0.1**

Significant p-values in bold.

## Discussion

We analysed impact of risk factors on mortality (500 days) in patients after first ever AF-associated CES or TIA in a population based stroke registry (LuSSt) particulary with regards to the effect of different OAC timings after FEIS. One-year mortality rate was 28.4%, which is in line with former study reports, where patients having a stroke associated with AF had a high risk of death in the year after FEIS [21].

Our data show that OAC non-treatment after AF-associated ischemic stroke is the main predictor for stroke mortality. Mortality was 5.4 times higher in patients not receiving OAC as compared to those that already received OAC in hospital. This fact again underlines the need for OAC treatment after CES [22]. Alarmingly, OAC-recommendation was only implemented in 71 out of 110 patients calling for improvement in outpatient care (e.g. by the implementation of special interventional programs) [23]. Interestingly, OAC initiation post discharge showed a trend (p = 0.1) towards lower mortality as compared to those that received OAC in hospital (HR 0.3; 95% CI 0.07-1.4) in multivariate analysis. Increased risk of bleeding under early treatment with OAC in more severe strokes might be one explanation [24]. Increased risk of ischemic stroke during first 30 days of treatment might also partly account for this [25]. In addition to OAC treatment, we found higher age and a higher mRS-score at discharge as independent predictors of mortality after CES. Recent data from the Canadian Stroke Network demonstrated association between older age and worse outcome after CES. Interestingly, there was no such association in those patients receiving therapeutic OAC treatment prior to stroke emphasizing the importance of OAC treatment in older patients [26]. It is well known that myocardial infarction is the leading cause of death in patients with cerebrovascular diseases [27]. We found CAD to be independently associated with mortality after CES. Antiplatelet agents such as Aspirin or Clopidogrel have shown to reduce ischemic events in patients with CAD [28]. Unfortunately, concomitant use of antiplatelet agents and OAC subsequently increases risk of bleeding [29]. Randomized trials have shown VKA to be as effective as a combination of VKA and Aspirin and more effective as Aspirin alone in CAD patients considering myocardial infarction [30]. Although recent observational studies showed decreased rate of MI in AF patients receiving Dabigatran as compared to Warfarin [31], certain subgroup analyses and metanalyses of randomized controlled trials demonstrated an increase of myocardial infarction [32,33]. However, data of end-point driven randomized controlled trials on NOACs are missing. Right now, OAC with VKA might remain important for CES patients having CAD. Recurrence rates were similar to those reported in previous trials [34,35]. Although being limited by small sample size, stroke recurrence did not influence survival in those patients receiving OAC, whereas there was a clear interaction in those patients that did not receive OAC. Clinical benefit of OAC in AF is again underlined.

Our study has strengths and limitations. Being based on registry data, study design was primarily descriptive and besides other confounding factors, quality of treatment of risk factors was not implemented, especially considering time of therapeutic range of VKA treatment. Furthermore we only analysed all-cause mortality. However, LuSSt is a population-based stroke registry without any age restrictions. Multiple overlapping methods were used to ensure nearly complete case ascertainment and high case completeness was achieved with regard to stroke mortality.

## Conclusion

In our study OAC non-treatment was the main predictor for mortality after FEIS. A later initiation of OAC treatment post hospital was not inferior to early initiation during hospital stay, however, one third of those patients who were recommended to be treated with OACs never received treatment. Special secondary preventive programs may increase the proportion of anticoagulated patients after stroke due to AF.
